# Large Language Models and Empathy: Systematic Review

**DOI:** 10.2196/52597

**Published:** 2024-12-11

**Authors:** Vera Sorin, Dana Brin, Yiftach Barash, Eli Konen, Alexander Charney, Girish Nadkarni, Eyal Klang

**Affiliations:** 1 Department of Radiology Mayo Clinic Rochester, MN United States; 2 Department of Diagnostic Imaging Sheba Medical Center Ramat Gan Israel; 3 The Faculty of Medicine Tel Aviv University Tel Aviv Israel; 4 DeepVision Lab Chaim Sheba Medical Center Tel Hashomer Israel; 5 Division of Data-Driven and Digital Medicine Icahn School of Medicine at Mount Sinai New York, NY United States; 6 The Charles Bronfman Institute of Personalized Medicine Icahn School of Medicine at Mount Sinai New York, NY United States

**Keywords:** empathy, LLMs, AI, ChatGPT, review methods, review methodology, systematic review, scoping, synthesis, foundation models, text-based, human interaction, emotional intelligence, objective metrics, human assessment, emotions, healthcare, cognitive, PRISMA

## Abstract

**Background:**

Empathy, a fundamental aspect of human interaction, is characterized as the ability to experience another being’s emotions within oneself. In health care, empathy is a fundamental for health care professionals and patients’ interaction. It is a unique quality to humans that large language models (LLMs) are believed to lack.

**Objective:**

We aimed to review the literature on the capacity of LLMs in demonstrating empathy.

**Methods:**

We conducted a literature search on MEDLINE, Google Scholar, PsyArXiv, medRxiv, and arXiv between December 2022 and February 2024. We included English-language full-length publications that evaluated empathy in LLMs’ outputs. We excluded papers evaluating other topics related to emotional intelligence that were not specifically empathy. The included studies’ results, including the LLMs used, performance in empathy tasks, and limitations of the models, along with studies’ metadata were summarized.

**Results:**

A total of 12 studies published in 2023 met the inclusion criteria. ChatGPT-3.5 (OpenAI) was evaluated in all studies, with 6 studies comparing it with other LLMs such GPT-4, LLaMA (Meta), and fine-tuned chatbots. Seven studies focused on empathy within a medical context. The studies reported LLMs to exhibit elements of empathy, including emotions recognition and emotional support in diverse contexts. Evaluation metric included automatic metrics such as Recall-Oriented Understudy for Gisting Evaluation and Bilingual Evaluation Understudy, and human subjective evaluation. Some studies compared performance on empathy with humans, while others compared between different models. In some cases, LLMs were observed to outperform humans in empathy-related tasks. For example, ChatGPT-3.5 was evaluated for its responses to patients’ questions from social media, where ChatGPT’s responses were preferred over those of humans in 78.6% of cases. Other studies used subjective readers’ assigned scores. One study reported a mean empathy score of 1.84-1.9 (scale 0-2) for their fine-tuned LLM, while a different study evaluating ChatGPT-based chatbots reported a mean human rating of 3.43 out of 4 for empathetic responses. Other evaluations were based on the level of the emotional awareness scale, which was reported to be higher for ChatGPT-3.5 than for humans. Another study evaluated ChatGPT and GPT-4 on soft-skills questions in the United States Medical Licensing Examination, where GPT-4 answered 90% of questions correctly. Limitations were noted, including repetitive use of empathic phrases, difficulty following initial instructions, overly lengthy responses, sensitivity to prompts, and overall subjective evaluation metrics influenced by the evaluator’s background.

**Conclusions:**

LLMs exhibit elements of cognitive empathy, recognizing emotions and providing emotionally supportive responses in various contexts. Since social skills are an integral part of intelligence, these advancements bring LLMs closer to human-like interactions and expand their potential use in applications requiring emotional intelligence. However, there remains room for improvement in both the performance of these models and the evaluation strategies used for assessing soft skills.

## Introduction

Empathy, a fundamental aspect of human interaction, can be characterized as the ability to experience the emotions of another being within oneself. The origin of the word “empathy” dates back to the 1880s, when Theodore Lipps determined the word “einfuhlung” (“in-feeling”) to describe the emotional appreciation of another’s feelings [1]. Empathy involves recognition of others’ feelings, the causes of these feelings, and the ability to participate in an emotional experience of an individual without becoming part of it [1].

Empathy is described as “the ability to see the world through someone else’s eyes,” having the ability to imagine what someone else is thinking and feeling in a given situation [2]. It is commonly understood to encompass cognitive and affective components: the ability to understand another’s feelings (cognitive empathy) and to experience emotions in response to others (affective empathy) [1,3].

In health care, empathy has an important role in patient care, improving patient satisfaction and treatment adherence. Empathy allows health care professionals to understand the emotional and psychological states of patients, fostering better communication and trust [4].

Large language models (LLMs) have demonstrated remarkable capabilities across various tasks, including text summarization, question-answering, and text generation [5]. There are numerous studies on potential applications in health care, as an educational tool and as a support tool in clinical work [6,7]. These models are already being integrated into practice. For instance, Epic has integrated GPT4 in its electronic health record software [8,9].

While LLMs have the potential to improve and automate some medical tasks, there are significant limitations to these models and their integration [10,11]. Despite the promising natural language processing capabilities, these models make errors and their performance in clinical tasks is challenging to evaluate on a large scale [12]. Many studies thus rely on multiple-choice questions assessment, which do not reflect real-world clinical applications [13]. These models can introduce bias [14], and can be susceptible to cyberattacks [15]. Some studies that evaluated these models for medical tasks suggested that despite impressive capabilities, LLMs lack empathy, a quality that is unique to humans and is imperative in health care [16-19].

Recent studies discuss and evaluate LLMs performance in tasks related to emotional intelligence, theory of mind, and empathy [20-25]. Some evidence suggests that these models may show aspects of cognitive empathy, including emotions recognition and providing supportive responses [17,26-28]. Furthermore, commercial LLM-based applications are being developed to offer emotional support to patients [29]. Given these developments, the aim of our study was to systematically review the literature on the capacity of LLMs in demonstrating empathy.

## Methods

We searched the literature on LLMs and empathy using MEDLINE, Google Scholar, PsyArXiv, medRxiv, and arXiv. Studies published between December 2022 and February 2024 were included. The search query was “((“large language models”) OR (llms) OR (gpt) OR (chatgpt)) AND ((empathy) OR (“emotional awareness”) OR (“emotional intelligence”) OR (emotion)) OR ((“social robots”) OR (“artificial emotional intelligence”) OR (“emotional artificial intelligence”) OR (“emotional chatbots”) OR (“affective computing”) OR (HRI) OR (“Human robot interaction”)).” We also searched the references lists of relevant studies, including some key studies from major medical journals, for any additional studies that may have been missed during the initial search.

The inclusion and exclusion criteria are detailed in Table 1. Two reviewers (VS and EK) independently performed the search and screened the titles and abstract of the articles resulting from the search. Differences in search results were resolved through discussion to reach a consensus. The reviewers then screened selected articles’ full text for final inclusion. Ultimately, 12 publications were included in this review. The results of the included studies including the LLMs used, performance in empathy tasks, and limitations of the models, along with studies’ publication details, authors, and other relevant information were systematically summarized in a table.

**Table 1:** Inclusion and exclusion criteria. This table outlines the inclusion and exclusion criteria applied to select studies for this review for evaluating empathy within large language models.

Criteria	Inclusion	Exclusion
Article type	Full-length original articles	Nonoriginal articles including but not limited to perspectives, opinions, and reviews
Language	English	Non-English
Focus of study	Articles that evaluated empathy within LLMs'^a^ outputs	Studies focusing only on emotion recognition or theory of mind, without explicit empathy evaluation
Model	Only LLMs^a^	Any other NLP^b^ algorithms

^a^LLM: large language model.

^b^NLP: natural language processing.

## Results

**Figure 1:**
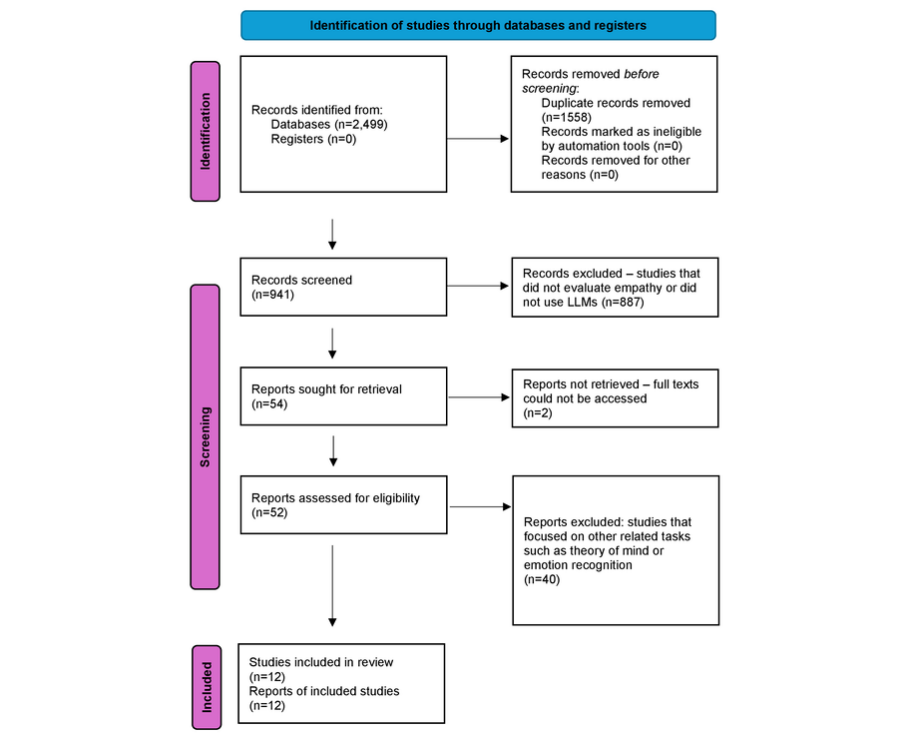
Flow diagram of the inclusion process based on the PRISMA (Preferred Reporting Items for Systematic Reviews and Meta-Analyses) guidelines.

Figure 1 presents a flow diagram of the screening and inclusion process. All 12 studies included in this review were published in 2023. Six studies compared ChatGPT-3.5 with other LLMs including GPT-4, versions of LLaMA, and fine-tuned chatbots. Six studies evaluated only ChatGPT-3.5. Seven studies evaluated empathy in ChatGPT in medical context. The results of the studies included are summarized in Table 2*.* This table provides a detailed summary of studies included in this review that evaluate aspects of empathy exhibited by large language models. The table outlines each study’s objectives, the specific large language model used key findings from the evaluations, sample sizes, and the methods used to assess empathy. It also highlights whether the reviewers were blinded to whether responses came from large language models or humans. The limitations of the LLMs as detailed in the different studies are detailed in Table S1 in Multimedia Appendix 1.

**Table 2:** Overview of studies evaluating empathy in large language models.

Study	Objective	LLM^a^	Key findings	Sample size	Methods for assessing empathy	Reviewers were blinded to LLM versus human responses
Webb [23]	Breaking bad news in emergency medicine	ChatGPT-3.5	ChatGPT facilitated realistic scenario design, active roleplay, and effective feedback through the application of the SPIKES^b^ framework for breaking bad news.	1 example	Simulating a patient role in an emergency department setting	No
Ayers et al [26]	Empathetic responses to patient questions	ChatGPT-3.5	ChatGPT responses were preferred by evaluators over physicians in 78.6% evaluations and were rated of significantly higher quality and empathy.	195 questions from social media	Health care professionals rated LLM and physician responses	Yes
Chen et al [27]	Simulating psychiatrists and patients in clinical psychiatric scenarios, and evaluating the expression of empathy in the interactions	Chatbots based on ChatGPT	ChatGPT-powered chatbots showed feasibility in simulating some aspects of empathy in psychiatric interactions, achieving a score of up to 3.43/4 when evaluated by humans for empathetic responses.	14 patients and 11 psychiatrists interacting with an LLM^a^	The participants interacted with the chatbots and scored their responses for empathy	No
Zhao et al [30]	Evaluate emotional dialogue understanding and generation and compare it with other supervised models	ChatGPT-3.5	Supervised models surpassed ChatGPT in emotion recognition. ChatGPT produced longer responses, but responses were also more specific to the context of the conversation compared with other models. When evaluating empathy within responses, humans preferred ChatGPT responses over EmpSOA in 54.33% of cases. When compared with MISC however, ChatGPT responses were preferred in 16% of cases.	100	3 readers rated the responses of different models for empathy	No
Yeo et al [31]	Emotional support for patients with cirrhosis and those with HCC^c^	ChatGPT-3.5	ChatGPT emulated empathetic responses and offered actionable recommendations for patients and caregivers.	4 prompts with 4 scenarios related to emotional support	The authors’ subjective description and assessment	No
Elyoseph et al [28]	Emotional awareness performance compared with the general population norms	ChatGPT-3.5	ChatGPT demonstrated significantly higher emotional awareness performance than general population norms, with improvements over time. LEAS^d^ scores were significantly higher than those of the general population (both men’s and women’s), on all the scales.	20 scenarios	LEAS compared with the general population norms. Two psychologists assessed the responses	No
Liu et al [32]	Fine-tuning an LLM to generate responses to patient questions	LLM based on LLaMA-65B; ChatGPT-3.5, GPT-4	GPT-4 and ChatGPT-3.5 outperformed the fine-tuned model. Both ChatGPT models and a fine-tuned LLaMA outperformed physician-generated responses.	10 questions	Physicians rated the responses of the chatbots and actual health care provider responses	Yes
Brin et al [33]	Evaluate ChatGPT and GPT-4 on USMLE^e^ soft-skill questions	ChatGPT, GPT-4	GPT-4 correctly answered 90% of questions, outperforming ChatGPT and humans.	80 multiple-choice USMLE soft skills questions	Correctness of responses and comparison of ChatGPT and GPT-4 performance to that of past users from the AMBOSS question bank	No
Huang et al [34]	Evaluate emotional responses to various situations	Text-davinci-003 (a variant of GPT-3), GPT-3.5-turbo, GPT-4, LLaMA-2 7B and LLaMA-2 13B	The different LLMs generally demonstrate appropriate emotional responses. None of the models exhibit strong alignment with human references.	400 situations	Measuring the change in LLMs’ different evoked emotions (overall 8 negative emotions) in response to situations compared with human benchmark	No
Chen et al [25]	Evaluate empathy, listening and comfort abilities of a fine-tuned LLM, compared with other LLMs	SoulChat, ChatGLM-6B, ChatGPT, MeChat	The fine-tuned model (SoulChat) outperformed the 3 other models in automatic metrics (ROUGE^f^ and BLEU^g^), and based on human evaluation. The mean empathy score ranged between 1.84-1.90 (on a scale of 0-2), compared with 1.62-1.65 for ChatGPT.	10,000 samples for automatic evaluation and 100 samples for manual evaluation	Automatic evaluation tools were used, as well as manual rating by three experts in psychology	No
Belkhir and Sadat [35]	Evaluate whether prompt engineering and an external emotion classifier can improve ChatGPT’s empathetic responses	ChatGPT	Prompt engineering and the use of an external emotion classifier improved ChatGPT performance, increasing accuracy for emotion labeling from 28.64% to 39.55%.	25,000 human dialogues	Labeling dialogues with emotion labels	No
Qian et al [36]	Evaluate the performance of LLMs in generating empathetic responses compared with other deep learning available models	GPT-3, GPT-3.5, ChatGPT	ChatGPT outperformed the other models in empathetic response generation, with a mean score of 4.64 (on a scale of 1-5).	100 dialogues for human evaluation	Automatic evaluation tools and three human raters	No

^a^LLMs: large language models.

^b^SPIKES: Setting up, Perception, Invitation, Knowledge, Emotions with Empathy, and Strategy or Summary.

^c^HCC: hepatocellular carcinoma.

^d^LEAS: Levels of Emotional Awareness Scale.

^e^USMLE: United States Medical Licensing Examination.

^f^ROUGE: Recall-Oriented Understudy for Gisting Evaluation.

^g^BLEU: Bilingual Evaluation Understudy.

Empathy is essential in medicine, particularly when breaking bad news to patients. It allows physicians to deliver difficult information in a manner that respects the patient’s emotions and perspective. Webb [23] used ChatGPT to simulate a role play for breaking bad news in the emergency department. The chatbot successfully set up a training scenario, role played as a patient and provided clear feedback through the application of the SPIKES (Setting up, Perception, Invitation, Knowledge, Emotions with Empathy, and Strategy or Summary) framework for breaking bad news [23]. In another study, Yeo et al [31] tested ChatGPT’s ability to provide emotional support to patients diagnosed with hepatocellular carcinoma, and their caregivers. ChatGPT was able to acknowledge the likely emotional response of the patient to their diagnosis. Furthermore, the chatbot provided clear and actionable starting points for a newly diagnosed patient and offered motivational responses encouraging proactive steps. For caregivers, ChatGPT provided psychological and practical recommendations [31].

Ayers et al [26] compared the quality and empathy of responses given by ChatGPT and physicians with 195 randomly drawn patient questions from a social media forum. The study found that patients preferred the chatbot’s responses over physician responses in 78.6% of cases. ChatGPT’s responses were rated significantly higher for both quality and empathy, while physician responses were 41% less empathetic than the chatbot responses. The authors noted that ChatGPT tended to provide more lengthy responses, which could potentially be erroneously associated with greater empathy. They concluded that the chatbot may have potential in aiding drafting responses to patient questions [26].

Another study also assessed empathy in chatbot’s responses to patient’s questions. Liu et al [32] developed a model based on a pretrained LLaMA-65B and finetuned to generate physician-like responses that are professional and empathetic. They evaluated the model on 10 actual patient questions in primary care and compared the responses with those generated by ChatGPT-3.5 and GPT-4, rating them based on empathy, responsiveness, accuracy, and usefulness. When evaluating empathy, GPT-4 and ChatGPT-3.5 outperformed their model. Interestingly, all language models outperformed physician-generated responses significantly [32].

Understanding and addressing patients’ emotions is fundamental in mental health. Chen et al [27] used ChatGPT-powered chatbots to simulate psychiatrists and patients in clinical psychiatric scenarios. The chatbots showed potential in simulating some aspects of empathy. However, they sometimes forgot initial instructions and repeated general empathy phrases too often. They also asked fewer in-depth questions about symptoms compared with physicians, potentially affecting their ability to fully understand the patient’s condition. When simulating patients, the chatbots reported symptoms inaccurately [27].

The Levels of Emotional Awareness Scale (LEAS) is a psychological tool that assesses an individual’s capacity to identify and describe emotions in themselves and others, a fundamental aspect of empathy [37]. Elyoseph et al [28] compared the LEAS score of ChatGPT to the general population norms. They found that ChatGPT demonstrated significantly higher emotional awareness performance. When repeating the test following 1 month interval, the chatbot’s performance further improved, almost reaching the maximum possible LEAS score. The authors propose that ChatGPT could be helpful for cognitive training of people with emotional awareness impairment, as well as for psychiatric assessment support [28].

Zhao et al [30] compared ChatGPT with supervised models in terms of emotional dialogue understanding and generation. The tasks they assessed included emotion recognition, emotion cause recognition, dialog act classification, empathetic response generation, and emotional support conversation. The authors found that while supervised models surpassed ChatGPT in emotion recognition, ChatGPT produced longer, more diverse, and context-specific responses, especially when interacting with users in negative emotional states. Interestingly, Zhao et al [30] also observed a repetitive pattern in ChatGPT’s empathy expressions, similar to the results described by Chen et al [27].

Brin et al [33] evaluated ChatGPT and GPT-4 on USMLE (United States Medical Licensing Examination) questions involving communication skills, ethics, empathy, and professionalism. They have used questions from the USMLE website and the AMBOSS question bank and compared the performance of the LLMs with the reported performance at the AMBOSS website. GPT-4 correctly answered 90% of questions, outperforming ChatGPT and humans [33]. Huang et al [34] evaluated emotional responses of 5 different LLMs to various situations designed to evoke emotions, The LLMs’ responses were compared with human responses collected from 1266 participants worldwide. The authors reported for each model the changes in emotion scores relative to human benchmarks. They conclude that the different LLMs generally demonstrate appropriate emotional responses. However, none of the models exhibited strong alignment with human references. GPT-3.5-turbo demonstrated the highest alignment in the scores after imagining being in the situations. The 13B version of LLaMA-2 exhibited the strongest comprehension of human emotions.

Chen et al [25] constructed an empathetic conversation dataset of over 2 million samples and used it to fine-tune an LLM to provide empathetic responses. Their finetuned LLM outperformed other LLMs including ChatGPT in responses’ coherence and relevancy, as well as empathy, helpfulness and safety.

Blekhir and Sadat [35] evaluated whether prompt engineering and external emotion classifier can enhance empathy in ChatGPT’s responses. The study evaluated 2 versions of ChatGPT: 1 incorporating user emotions with an emotion classifier and another adapting to emotions without external tools. They evaluated these versions against the standard ChatGPT, demonstrating that tailored emotional responses significantly improve ChatGPT’s empathetic capabilities [35].

Qian et al [36] evaluated ChatGPT compared with other deep-learning models trained for empathetic interactions. They also propose 3 improvement methods including semantically similar in-context learning, 2-stage interactive generation, and combination with knowledge base. These methods improved the quality of responses generated by ChatGPT, which outperformed other models evaluated [36].

Lee et al [38] used Chain-of-Empathy prompting to reason emotion and situational factors that may assist the model to infer the emotional experience. They evaluated GPT-3.5 and compared 4 unique prompts that used Chain-of-Empathy in generating empathetic responses to Reddit posts. The Chain-of-Empathy strategy resulted in improved the model’s empathy expression [38].

## Discussion

### Principal Findings

This review shows that LLMs demonstrate aspects of cognitive empathy, including recognition of emotions, and generation of emotionally supportive responses. Most studies focused on LLMs’ performance in medical contexts, assessing their ability to provide empathetic responses in clinical and nonclinical scenarios. Notable, LLMs were reported in the majority of the studies to perform comparably or even surpass human responses in certain empathy-related tasks. The review also identifies limitations, including the subjective nature of empathy evaluation, the risk of overestimating empathy due to lengthier responses, and the models’ inherent lack of emotional experience.

### Empathy and Social Intelligence in LLMs

LLMs have shown impressive abilities in semantic understanding and logical reasoning [5]. The ability of LLMs to emulate empathy, especially cognitive empathy, mirrors the growing body of research demonstrating that artificial intelligence can replicate certain aspects of social intelligence. This review supports the idea that LLMs may demonstrate some abilities that resemble social intelligence. Theory of mind involves the understanding of others’ thoughts and emotions, and predicting or explaining their behaviors based on these inferences. This concept is fundamental to social interactions, and it is a complex task, as it involves understanding not just the literal meaning of words in a conversation, but the underlying intentions, beliefs, and emotions [39]. Several studies evaluated LLMs on theory of mind tasks, with varied performance, depending on the tasks and the models used [20-22,39,40].

### Cognitive Versus Affective Perspectives

The definition of empathy varies among researchers and practitioners in social sciences [1]. One of the debates is whether it is a cognitive or affective concept, and most definitions of empathy include both [1]. Cognitive empathy involves the ability to understand another’s feelings, closely related to theory of mind [3]. Affective empathy relates to experiencing emotions in response to an emotional stimulus [1]. The ability of LLMs to demonstrate empathy in various fields as highlighted in this review, seems to align more with the cognitive aspect. It is nevertheless surprising that in some cases the LLM outperformed humans in empathy-related tasks.

Research suggests that cognitive and affective empathy are distinct. For instance, people with autism often struggle with cognitive empathy but have normal levels of affective empathy, while psychopathic individuals typically show the reverse pattern [3]. Neurological studies demonstrated distinct brain regions associated with each type of empathy, which further supports this notion [41,42]. It is worth questioning if demonstrating cognitive empathy alone is sufficient, or whether affective empathy is imperative for achieving human-like emotional intelligence.

Historically, empathy has been viewed as a uniquely human trait, with definitions focused on interactions between humans [1]. The complexity of empathy, influenced by personality, culture, and context, has led to ambiguous definitions of the term [1,20]. Empathy exhibited by AI fundamentally differs from human empathy because an algorithm does not engage in a human’s emotional experience. Consequently, human-centric definitions of empathy may not apply to LLMs. This warrants a reevaluation of how empathy is measured. The question arises whether observable responses alone can be considered empathetic if they meet human expectations or preferences. If humans cannot distinguish between responses generated by humans and LLMs, or if they prefer AI-generated responses as demonstrated in the study by Ayers et al [26], perhaps emulating such empathy may be sufficient.

### Implications in Health Care

Numerous studies support the remarkable performance of LLMs in clinical reasoning [6,7]. These models can be applied to enhance the medical care patients receive, while decreasing the workload of health care providers [43]. Yet, empathy is a key factor in patient care. Empathy in health care communication is linked to improved patient satisfaction, adherence to treatment plans, and better outcomes [4]. It allows for a more nuanced understanding of patients’ emotional states and experiences, facilitating more compassionate and person-centered care. As such, the ability of LLMs to integrate empathy can significantly enhance the role of AI in health care, for both patients and health care providers.

Empathy in health care aligns more with cognitive rather than affective empathy, involving the ability to understand the pain and suffering of patients, and the capability to communicate this understanding [44,45]. Using this perspective, tools like the Jefferson Scale can assess empathy within health care settings [45,46]. There may be scenarios where LLMs might demonstrate more fitting empathy, especially in contexts where cognitive empathy is predominant. However, the lack of standardized methods for assessing empathy in LLMs, as also seen in this review, challenges the ability to compare their empathetic capacities across different models and tasks.

Furthermore, LLMs’ empathy is influenced by cultural factors, norms, and contexts, affecting how empathy is perceived by individuals from diverse backgrounds. Awareness of interacting with an AI could bias perceptions of empathy, potentially undermining its authenticity [47]. Conversely, LLMs possess the potential to overcome cultural divides, offering empathetic responses appropriate across various backgrounds.

### Limitations

This review has several limitations. First, as all but 1 study evaluated empathy based on subjective assessment, we could not perform a meta-analysis. Second, we only assessed studies directly discussing empathy, while there are many more that evaluate theory of mind tasks that are closely related to “cognitive” empathy. Third, all studies assessed ChatGPT-3.5, and only 1 study evaluated a model based on LLaMA and GPT-4. This can potentially limit the generalizability of findings to other LLMs. It is possible that alternative LLMs may present different empathy characteristics. Furthermore, LLMs are evolving fast, and possibly newer LLMs will present higher cognitive-like abilities.

### Conclusion

To conclude, this review demonstrates that LLMs exhibit elements of cognitive empathy, being able to recognize emotions and provide emotionally supportive responses in various contexts. Given that social skills are foundational to the concept of “intelligence,” further research is warranted to further develop that aspect in AI. The ability to simulate empathetic responses could enhance patient experiences, improving patient satisfaction and adherence to treatment plans. However, there remain critical questions regarding whether LLMs’ cognitive empathy is sufficient in scenarios that require deeper emotional engagement. Ultimately, as we continue to refine these models, we approach closer to bridging the gap between artificial and human-like interactions, opening opportunities for empathetic AI applications.

## Appendix

Multimedia Appendix 1 Supplemental Table 1. Limitation of Large Language Models Detailed in the Studies Included.

Multimedia Appendix 2 PRISMA (Preferred Reporting Items for Systematic Reviews and Meta-Analyses) checklist.

## Abbreviations

LEAS: Levels of Emotional Awareness Scale
LLM: large language model
SPIKES: Setting up, Perception, Invitation, Knowledge, Emotions with Empathy, and Strategy or Summary
USMLE: United States Medical Licensing Examination

## Data Availability

Data extracted from the studies are available in Table 2, and upon request from authors.
