# Supplementary material for: Large Language Models and Empathy: Systematic Review
Source: J Med Internet Res. 2024 Dec 11;26:e52597. doi: 10.2196/52597 (PMC11669866; doi:10.2196/52597)
Supplement: Multimedia Appendix 1 [file jmir_v26i1e52597_app1.docx]

**Supplemental Table 1. Limitation of Large Language Models Detailed in the Studies Included**

| Study | Limitations of LLMs^a^ |
| --- | --- |
| Webb [1] | Specific limitations were not discussed. General limitations discussed include limited training dataset, performance being influenced by prompts design, potential for inaccurate responses and inability to convey parts of human communications such as eye contact, pausing to listen, and tone. |
| Ayers et al [3] | Not specifically discussed. ChatGPT tended to provide more lengthy responses, which could potentially be erroneously associated with greater empathy. The study did not assess the chatbot responses for accuracy or fabricated information. |
| Chen et al [4] | The chatbots sometimes forgot initial instructions and showed excessive repetition of general empathy phrases. In addition, they asked fewer in-depth questions about the symptoms compared with human doctors, potentially affecting their ability to fully understand the patient's condition. The patient chatbots reported symptoms inaccurately. |
| Zhao et al [6] | ChatGPT generated longer responses, and deviated from reference responses when evaluated based on word overlap metrics. It also demonstrated limited understanding of some labels. ChatGPT did not adhere to the same guidelines to determine emotions as were used for the annotated data for the supervised models. |
| Yeo et al [7] | In total, ChatGPT provided comprehensive answers in less than 50% of questions. |
| Elyoseph et al [5] | Not specifically discussed. |
| Liu et al [8] | One of the reviewer indicated “excessive empathy” in the fine-tuned LLM model. Responses generated by GPT models were considered too lengthy and required a relatively high reading level. |
| Brin et al [9] | ChatGPT was inconsistent and revised its answers when given the opportunity. GPT-4 on the other hand never revised its initial answers. |
| Huang et al [10] | The LLMs generally tended to exhibit higher negative scores compared with human subjects |
| Chen et al [2] | The authors discuss the complexity of empathy, while different users have different expectations from the model, specifically when discussing tense emotions |
| Belkhir et al [11] | N/A |
| Qian et al [12] | The authors do not explicitly detail LLMs limitations, but address the complexity of empathy as a concept and the influence of individual personalities, backgrounds and cultures on the expression of empathy. |

References

1. Webb JJ. Proof of Concept: Using ChatGPT to Teach Emergency Physicians How to Break Bad News. Cureus. 2023. doi: 10.7759/cureus.38755.

2. Chen Y, Xing X, Lin J, Zheng H, Wang Z, Liu Q, et al., editors. Soulchat: Improving llms’ empathy, listening, and comfort abilities through fine-tuning with multi-turn empathy conversations. Findings of the Association for Computational Linguistics: EMNLP 2023; 2023.

3. Ayers JW, Poliak A, Dredze M, Leas EC, Zhu Z, Kelley JB, et al. Comparing Physician and Artificial Intelligence Chatbot Responses to Patient Questions Posted to a Public Social Media Forum. JAMA Internal Medicine. 2023;183(6):589. doi: 10.1001/jamainternmed.2023.1838.

4. Chen S, Wu M, Zhu KQ, Lan K, Zhang Z, Cui L. LLM-empowered Chatbots for Psychiatrist and Patient Simulation: Application and Evaluation. arXiv preprint arXiv:230513614. 2023.

5. Elyoseph Z, Hadar-Shoval D, Asraf K, Lvovsky M. ChatGPT outperforms humans in emotional awareness evaluations. Frontiers in Psychology. 2023;14. doi: 10.3389/fpsyg.2023.1199058.

6. Zhao W, Zhao Y, Lu X, Wang S, Tong Y, Qin B. Is ChatGPT Equipped with Emotional Dialogue Capabilities? arXiv preprint arXiv:230409582. 2023.

7. Yeo YH, Samaan JS, Ng WH, Ting P-S, Trivedi H, Vipani A, et al. Assessing the performance of ChatGPT in answering questions regarding cirrhosis and hepatocellular carcinoma. Clinical and Molecular Hepatology. 2023;29(3):721-32. doi: 10.3350/cmh.2023.0089.

8. Liu S, McCoy AB, Wright AP, Carew B, Genkins JZ, Huang SS, et al. Leveraging Large Language Models for Generating Responses to Patient Messages. 2023. doi: 10.1101/2023.07.14.23292669.

9. Brin D, Sorin V, Vaid A, Soroush A, Glicksberg BS, Charney AW, et al. Comparing ChatGPT and GPT-4 performance in USMLE soft skill assessments. Scientific Reports. 2023;13(1). doi: 10.1038/s41598-023-43436-9.

10. Huang J-t, Lam MH, Li EJ, Ren S, Wang W, Jiao W, et al. Emotionally numb or empathetic? evaluating how llms feel using emotionbench. arXiv preprint arXiv:230803656. 2023.

11. Belkhir A, Sadat F, editors. Beyond information: Is chatgpt empathetic enough? Proceedings of the 14th International Conference on Recent Advances in Natural Language Processing; 2023.

12. Qian Y, Zhang W-N, Liu T. Harnessing the Power of Large Language Models for Empathetic Response Generation: Empirical Investigations and Improvements. arXiv preprint arXiv:231005140. 2023.
